# EFEMP2 suppresses epithelial-mesenchymal transition via Wnt/β-catenin signaling pathway in human bladder cancer

**DOI:** 10.7150/ijbs.35541

**Published:** 2019-08-08

**Authors:** Qiang Zhou, Song Chen, Mengxin Lu, Yongwen Luo, Gang Wang, Yu Xiao, Lingao Ju, Xinghuan Wang

**Affiliations:** 1 Department of Urology, Zhongnan Hospital of Wuhan University, Wuhan, China; 2 Department of Biological Repositories, Zhongnan Hospital of Wuhan University, Wuhan, China; 3 Human Genetics Resource Preservation Center of Hubei Province, Wuhan, China; 4 Laboratory of Precision Medicine, Zhongnan Hospital of Wuhan University, Wuhan, China; 5 Medical Research Institute, Wuhan University, Wuhan, China; 6 Urological Clinical Research Center of Laparoscopy in Hubei Province, Wuhan, China

**Keywords:** EFEMP2, bladder cancer, epithelial-mesenchymal transition, Wnt/β-catenin signaling pathway

## Abstract

Epidermal growth factor-containing fibulin-like extracellular matrix protein 2 (EFEMP2), an extracellular matrix protein, is highly associated with tumor invasion and metastasis. However, influenced by the tumor microenvironment, EFEMP2 played different roles in different tumors. The current study focused on exploring the role of EFEMP2 in bladder cancer (BCa). The results suggested that the expression of EFEMP2 was significantly higher in normal tissues and cells compared with BCa samples and cells. And we found a negative correlation between EFEMP2 expression and high tumor stage, high tumor grade, patients with low EFEMP2 expression had a much poorer survival than those patients with high EFEMP2 expression. The multivariate analysis revealed that low EFEMP2 expression was a high-risk predictor of BCa survival. Furthermore, cell proliferation, migration and metastasis can be obviously affected by the changes of EFEMP2 expression both in vitro and in vivo.

Our results also turned out that knockdown of EFEMP2 could significantly reduce the epithelial marker (E-cadherin), increase mesenchymal markers (N-cadherin, Vimentin, Snail and Slug) as well as the key factors of Wnt/β-catenin signaling pathway (β-catenin, c-Myc and cyclin D1). The reversed results were found in the EFEMP2 overexpression cells. Importantly, the related expression changes of epithelial-mesenchymal transition (EMT) markers and Wnt/β-catenin signaling pathway factors induced by EFEMP2 upregulation or downregulation can be rescued using LiCl or XAV939. Collectively, our observations revealed that EFEMP2 is a blocker of tumor progression and metastasis in BCa.

## Introduction

Bladder cancer (BCa) is one of the most common malignant carcinomas, with an estimated 549,393 new cases and 199,922 deaths per year in the world 1. Of all patients with BCa diagnosed, approximately 70% of those are non-muscle-invasive diseases at initial exhibition. Transurethral resection of tumor and intravesical chemotherapy have been considered as the standard for initial plan of action when treating non-muscle-invasive tumors. However, up to 70% will experience tumor recurrence and as many as 10%-30% ultimately lead to invasive tumors after management. For muscle-invasive BCa, the optimal method of surgery is radical cystectomy. About half of another 30% invasive diseases will live a long-time disease-free life after received radical cystectomy, in contrary, the other 50% patients will lead to death soon 2. Since patients with BCa display a heterogeneous group concerning their prognosis, patients always needing long-term follow-up with cystoscopy and computed tomography (CT) scan in case of relapse 3. As a result, the management costs of BCa seem considerable higher than other cancer. Therefore, effective methods of accurately predicting the prognosis of BCa is imperative requirement 4, 5.

Epidermal growth factor-containing fibulin-like extracellular matrix protein 2 (EFEMP2), also known as fibulin-4, is a member of the fibulin family, which was made up of fibulin-1, 2, 3, 4, 5, 6 and 7 6. The family is a group of extracellular matrix proteins, firstly discovered in 1989 7, which play an important role in regulating the interaction between cells and cells, cells and extracellular matrices 8, and reports have shown that fibulin protein is involved in the occurrence and development of tumors 9. EFEMP2 is necessary for elastic fiber formation and connective tissue development 10. Influenced by the tumor microenvironment, EFEMP2 plays different roles in different tumors 11, 12. In cervical carcinoma, high EFEMP2 expression is associated with lymph node metastasis and poor prognosis in patients, and high expression of EFEMP2 may promote angiogenesis 13. Inversely, the expression of EFEMP2 in breast cancer tissues was significantly lower than that in adjacent tissues, and its expression was negatively correlated with breast cancer tumor grade, suggesting that EFEMP2 can be used as an independent predictor of breast cancer prognosis 14. However, whether there is also a difference in EFEMP2 expression in bladder cancer and whether it plays a biological role in bladder cancer remains to be uncovered.

Epithelial-mesenchymal transition (EMT) is a multi-stage process that plays an important role in embryonic development and participates in the formation of various germ layers and organs 15, 16. EMT is characterized by the disappearance of the polarity of epithelial cells and adhesion proteins between cells, the acquisition of phenotype and exercise capacity of the mesenchymal cell 17. Growing evidences showed that this normal physiological procedure also exists in various pathogenic environments such as tumor invasion and metastasis 18. Many pathways can influence the EMT process, including the Wnt-catenin signaling pathway 19, 20. The Wnt/β-catenin signaling pathway is involved in the regulation of tumor signal transduction, and its main role is to promote the proliferation, differentiation and metastasis of tumor cells 21.

In the current study, we first depicted that the expression of EFEMP2 was significantly associated with tumor stage and grade in human BCa, and low EFEMP2 expression was an independent predictive factor of the relapse, progression and metastasis. Furthermore, overexpression of EFEMP2 exerted a negative impact on proliferation, migration and metastasis both in vitro and in vivo experiment, and to a certain extent Wnt/β-catenin signaling pathway may play a role in these processes.

## Materials and methods

### Microarray data, data processing and screening of differentially expressed genes

Microarray data GSE40355 contains 16 BCa tissues and 8 normal bladder tissues was downloaded from Gene Expression Omnibus (GEO) database (*http://www.ncbi.nlm.nih.gov/geo/*) in the National Center for Biotechnology Information (NCBI). Expression matrix was formed with the raw counts of each RNA of each sample. EdgeR package in R language was used to normalized expression matrix, then make comparisons between bladder tumor tissues and paracancerous normal specimens to screen the differentially expressed RNAs. |log2FC|>1 and FDR<0.05 were used as the threshold. The expression level of differentially expressed RNAs was log2(*+1)-transformed for further analysis. Cluster analysis and heatmap were conducted on the differentially expressed RNAs in order to better differentiate normal specimens from BCa specimens.

### Patients and bladder tissue specimens

A total of 21 pairs fresh BCa, adjacent tissues and another 25 BCa samples for total RNA isolation were stored in liquid nitrogen when after surgery immediately. Formalin-fixed, paraffin-embedded specimens of BCa (n = 231) and paracancerous (n = 39) were collected for immunohistochemical (IHC) from November 2010 to September 2013. All the samples were obtained from patients who firstly received surgery at Zhongnan Hospital of Wuhan University with patients' informed consent. Patients those who had preoperative chemotherapy and had history of other cancer were not accepted. Of all the 231 patients, the average age was (65.4 ± 10.87) years, included 177 males and 54 females. The pathology results represented that 192 cases were Ta, T1 and 39 members were T2, T3, T4. And 141 cases were papillary urothelial neoplasm of low malignant potential (PUNLMP) and low grade, the other 81 cases were high grade. The tumor stage and grade in all patients with bladder cancer were diagnosed according to 2009 TNM staging system and 2004 World Health Organization grading system, respectively. All patients under a regular follow-up, and the deadline of follow-up is December 31, 2016. During the period of follow-up, 9 patients were lost. The research was carried out under the permission of the Ethics Committee of Zhongnan Hospital of Wuhan University (approval number: 2015029).

### Cell lines and cell culture

A nontumorous immortalized bladder cell line, SV-HUV-1 and three BCa cell lines (T24, UM-UC-3 and RT4) were generously provided by the Stem Cell Bank, Chinese Academy of Sciences in Shanghai, China. Another two BCa cell lines, J82 and SW780, were purchased from the Procell Co., Ltd. in Wuhan, China. The verification of the all cell lines were finished by the China Centre for Type Culture Collection in Wuhan, China. SV-HUV-1, T24 and RT4 cells were cultured in RPMI-1640 medium (Gibco, China), UM-UC-3 cells were cultured in high-glucose DMEM medium (Gibco, China), J82 cells were cultured in MEM medium (Gibco, China), and SW780 cells were cultured in L-15 medium (Gibco, China) supplemented with 10% fetal bovine serum (FBS). All the cells were grown in a humidified environment at 37 °C with a condition of 5% CO_2_ and 95% air excepted for SW780 cells' 100% air.

### RNA extraction, reverse transcription and quantitative real-time PCR (qRT-PCR)

Total RNA was extracted from BCa cells and bladder tissues using HiPure Total RNA Mini Kit (Cat. #R4111-03, Magen, China) according to the manufacturer's instruction. The quality of isolated RNA was evaluated using NanoPhotometer (Cat. #N60, Implen, Germany). The reverse transcription process was carried out with the ReverTra Ace qPCR RT Kit (Toyobo, China). A total 20 μl-volume reaction system, which contained 1 μl cDNA, 1 μl of each primer, 10 μl iTaqTM Universal SYBR® Green Supermix (Bio-Rad, USA), and 7 μl DNAse/RNAse-free water, was performed in triplicates using a Bio-Rad iCycler (Cat. #CFX96). The expression of each gene was normalized to GAPDH expression. The primer sequences were listed as follows: EFEMP2: 5′-AAGAGCCCGACAGCTACAC-3′, 5′-AGGGATGGTCAGACACTCGTT-3′; E-cadherin: 5′-CGAGAGCTACACGTTCACGG-3′, 5′-GGGTGTCGAGGGAAAAATAGG-3′; N-cadherin: 5′-TGCGGTACAGTGTAACTGGG-3′, 5′-GAAACCGGGCTATCTGCTCG-3′; Vimentin: 5′-AGTCCACTGAGTACCGGAGAC-3′, 5′-CATTTCACGCATCTGGCGTTC-3′; Slug: 5′- TGTGACAAGGAATATGTGAGCC-3′, 5′-TGAGCCCTCAGATTTGACCTG-3′; Snail: 5′-ACTGCAACAAGGAATACCTCAG-3′, 5′-GCACTGGTACTTCTTGACATCTG-3′; β-catenin: 5′-AAAGCGGCTGTTAGTCACTGG-3′, 5′-CGAGTCATTGCATACTGTCCAT -3′; cyclin D1: 5′-GCTGCGAAGTGGAAACCATC-3′, 5′-CCTCCTTCTGCACACATTTGAA-3′; c-Myc: 5′-GTCAAGAGGCGAACACACAAC-3′, 5′-TTGGACGGACAGGATGTATGC-3′; GAPDH: 5′-GGAGCGAGATCCCTCCAAAAT-3′, 5′-GGCTGTTGTCATACTTCTCATGG-3′.

### Plasmid construction

A 1322-bp fragment of EFEMP2 was amplified from cDNA of T24 cells using the forward primer 5'-CGGGGTACCGCCACCATGCTCCCCTGCGCCTCCT-3' and the reverse sense 5'-GCGCCCG CTCGAGTCAGAAGGTGTAGGCCCCTACA-3', and the products were directly cloned into 2xFlag-pcDNA3 empty vector. The DNA sequence was identified by sequencing.

### Cell transfection and stable cell lines construction

The small interfering RNA (siRNA) of EFEMP2 and the negative-control siRNA were synthesized by GenePharma in Suzhou, China. The sense sequence of EFEMP2-siRNA-1 was 5'-GCAAUGCACUGACGGAUAUTT-3', EFEMP2-siRNA-2 was 5'-GCUGACAGUCAGCAGUAUATT-3', and the negative control sense sequence was 5′-UUCUCCGAACGUGUCACGUTT-3′. The siRNA and plasmid transfection of BCa cells were accomplished via Lipofectamine 2000 (Cat. #11668-019, Invitrogen Life Technologies, USA) following the manufacturer's protocol. After 48 hours transfection, the BCa cells were harvested for assessment of alterations of mRNAs and proteins by immunofluorescence staining, qRT-PCR and Western blot. The Wnt/β-catenin signaling pathway inhibitor XAV-939 (10 μmol/l, Cat. # X3004, Sigma, USA) and activator LiCl (20 mmol/l, Cat. # 213233, Sigma, USA) treated cells for 24 hours when after 24 hours siRNA or plasmid transfection. For protein stability analysis, cells were treated with 100 μg/ml cycloheximide (MCE, HY-12320) and harvested after different incubation times.

For the construction of stable cell lines, both lentiviral-EFEMP2-oeRNA (LV-EFEMP2 oe) and lentiviral-vector-oeRNA (LV-V) (purchased from GenePharma in Suzhou, China) were applied to transfect T24 cells, cells were incubated for 24 hours, then treated with 2 μg/ml-puromycin (Sigma, USA) medium at least 7 days to establish stable EFEMP2 over-expression cell line.

### Methyl thiazolyl tetrazolium (MTT), clonogenic survival and transwell migration assay

For MTT (MO, USA) assay, after 48 hours management, digested BCa cells were collected, 3000 or 5000 cells per 200 μl suspension medium (3000 cells for T24 and UM-UC-3, 5000 cells for J82) planted on 96-well plates with 6 repeated wells, then 5mg/ml-20 μl MTT was added into each well every 24, 48, 72, 96 and 120 hours, the mixture was incubated for 4h at 37 °C. After removing the mixture, 200 μl DMSO was applied to dissolve the precipitates. The absorbance of all wells was assessed at 490 nm by a microplate reader (Cat. #SpectraMax M2, Molecular Devices, USA).

For clonogenic survival assay, after 48 hours treatment, digested BCa cells were harvested, 1500 cells were seeded in triplicates on 6-well plates per well with 2 ml medium to grow into colonies for nearly 15 days. The medium was discarded, PBS was used to wash the colonies. Then the colonies were fixed in 4% paraformaldehyde (PFA) for approximately 30 min, stained with 0.1% crystal violet. The colonies were counted, photographed and statistically analyzed.

Briefly for transwell migration assay, we used a 24-well plate transwell chamber system (Corning, USA). In the upper chamber (Corning, USA), 3×104 cells were suspended in 200 μl serum-free medium in triplicates, while 600 μl 10% FBS medium was added in the lower chamber to tempt cell migration action. After incubating 24 hours, cotton swab was applied to wipe out the cells in the upper chamber, the same as the colonies, those cells who entered into the other side of membrane were fixed in 4% PFA for 30 min, stained with 0.1% crystal violet. The stained chambers were left to dry and were photographed using an optical microscope.

### Flow cytometry analysis for the alterations of cell cycle

After transfection for 48 h, BCa cells were harvested and washed by cold PBS. Then, the cells were resuspended with 1× DNA Staining Solution containing propidium iodide and permeabilization solution (Multisciences, China) in the dark. The sample was analyzed by flow cytometry analysis (Cat. #FC500, Beckman, USA) after incubation at 37°C for 30 min.

### Protein isolation and Western blot

After treatment, BCa cells were collected into a 1.5 ml and lysed in RIPA buffer solution supplemented with 1μl phosphatase inhibitor (Sigma-Aldrich, USA) and 1 μl protease inhibitor every 50 μl volume. The samples were placed on ice for 30 min with discontinuous vortexing. The lysates were centrifuged at 13000 rpm for 15 min at 4 °C. The supernatant of lysates was harvested and protein concentration was quantified using Bradford Protein Assay kit (Bio-Rad, Germany). After denatured at 100 °C for 10 min, the extracted protein samples were loaded and resolved by 6-15% SDS-PAGE, transferred onto PVDF membranes (Millipore, USA) which were when blocked with 5% non-fat milk for 2 hours at room temperature. Membranes were incubated with primary antibodies overnight at 4 °C, followed by secondary antibodies incubation for another 2 hours at room temperature the next day, and bands were developed exposed by an enhanced chemiluminescence (ECL) kit (Bio-Rad, USA) via a Bio-Rad ChemiDoe XRS^+^ Imaging System (Bio-Rad, USA).

The following antibodies were utilized in the Western blot: anti-EFEMP2, 1:1000 (ab125073, Abcam); anti-E-cadherin, 1:1000 (3195S, Cell Signaling); anti-N-cadherin, 1:1000 (13116T, Cell Signaling); anti-Vimentin, 1:1000 (5741S, Cell Signaling); anti-Slug, 1:1000 (7585P, Cell Signaling); anti-snail, 1:1000 (3879T, Cell Signaling); anti-β-catenin, 1:1000 (610154, BD Biosciences); anti-c-Myc, 1:1000 (ab32072, Abcam); anti-cyclin D1, 1:1000 (ab134175, Abcam), and anti-GAPDH, 1:2000 (sc-365062, Santa Cruz) was playing the role as a loading control.

### Immunofluorescence, hematoxylin and eosin (HE) and immunohistochemistry (IHC) staining

For the immunofluorescence staining, the treated BCa cells were gained and plated onto 12 mm coverslips. After incubating 24 hours, PBS was used to wash the coverslips which then fixed with 4% PFA for 30 min. Thereafter, the rest processes were performed by Biofavor Biotech Ltd. in Wuhan, China. A Confocal microscope system (Nikon C2^+^ Confocal Microscope, Japan) was utilized to observe and picture the final results.

For the HE staining, briefly, after baking the tissue sections (5 μm) were invaded into xylene for dewaxing followed by rehydration with graded alcohol (100%, 100%, 95%, 80% and 70%) solutions. Then 10% hematoxylin was used for variegation, 1% eosin contained 0.2% glacial acetic acid solution was applied to differentiate the cytoplasm for only seconds. Thereafter, the slides were washed, dehydrated with another graded alcohol (80%, 95%, 100% and 100%). Finally, the clarity with xylene was before the sealing with neutralresinsize.

And for the IHC, the procedures of dewaxing and rehydration were the same as HE staining. Then, the tissue pieces were boiled in citrate buffer (pH 6.0) at 100 °C for 15 min for the antigen retrieval. Primary antibody (anti-EFEMP2, 1:100, ab125073, Abcam) was added after blocking for 10 min at room temperature with 3.0% hydrogen peroxide (H_2_O_2_). The slides were exposed to secondary antibody for 30 min at room temperature after incubating overnight at 4 °C. Lastly, the sections were incubated with DAB chromogen and then counterstained with hematoxylin. The IHC assessment was completed by two pathologists who were uninformed to clinical outcomes. The scoring of EFEMP2 expression was defined as 0, 1, 2 or 3 according to staining of intensity, and the ultimate staining score were categorized as low (0, 1), and high (2, 3).

### Xenograft model and pulmonary metastasis model

Specific-pathogen-free (SPF) male BALB/c-nude mice (3-week-old) were purchased from Beijing Vital River Laboratory Animal Technology Co., Ltd. (Beijing, China). After a week adaptive raise at the laboratory animal facility of Zhongnan Hospital of Wuhan University in a SPF environment. We randomly assigned mice to the control group and the experimental group. For the xenograft model, 1×106 T24 LV-V or LV-EFEMP2 oe cells suspended in 0.2 ml of serum-free medium were subcutaneously injected into 4-week-old BALB/c-nude mice (4 mice per group). The tumor size was measured with vernier scale every week for 7 weeks (tumor volume = length×width^2^×0.5 mm^3^), and the weight of each tumor was also assessed at the 49th day when the mice sacrificed. The tumors were fixed in 4% PFA and then analyzed by HE and IHC stainings. Also, for the pulmonary metastasis model, 1×10^6^ T24 LV-V or LV-EFEMP2 oe cells were injected into the tail vein of mice, and the fluorescence of BCa cells pulmonary metastasis was observed by Fusion FX7 Spectra Imaging system (Vilber, France) after 5-week feeding. The lung tissues were analyzed by HE.

### Statistical analysis

The SPSS software package (version 19.0) was used for all statistical analysis. The statistical significance of differences was compared using the chi-square test and Student's t test as appropriate. Kaplan-Meier estimates for overall survival (OS), metastasis-free survival (MFS), progress-free survival (PFS) and disease-free survival (DFS) were compared using the log rank test. For the univariate and multivariate analyses, the Cox proportional hazards regression model was used and summarized with the hazard ratio (HR) and 95% confidence interval (CI). A two-side and P values of < 0.05 were considered statistically significant.

## Results

### Differentially expressed RNA screening between bladder cancer and normal specimens

**Figure 1:**
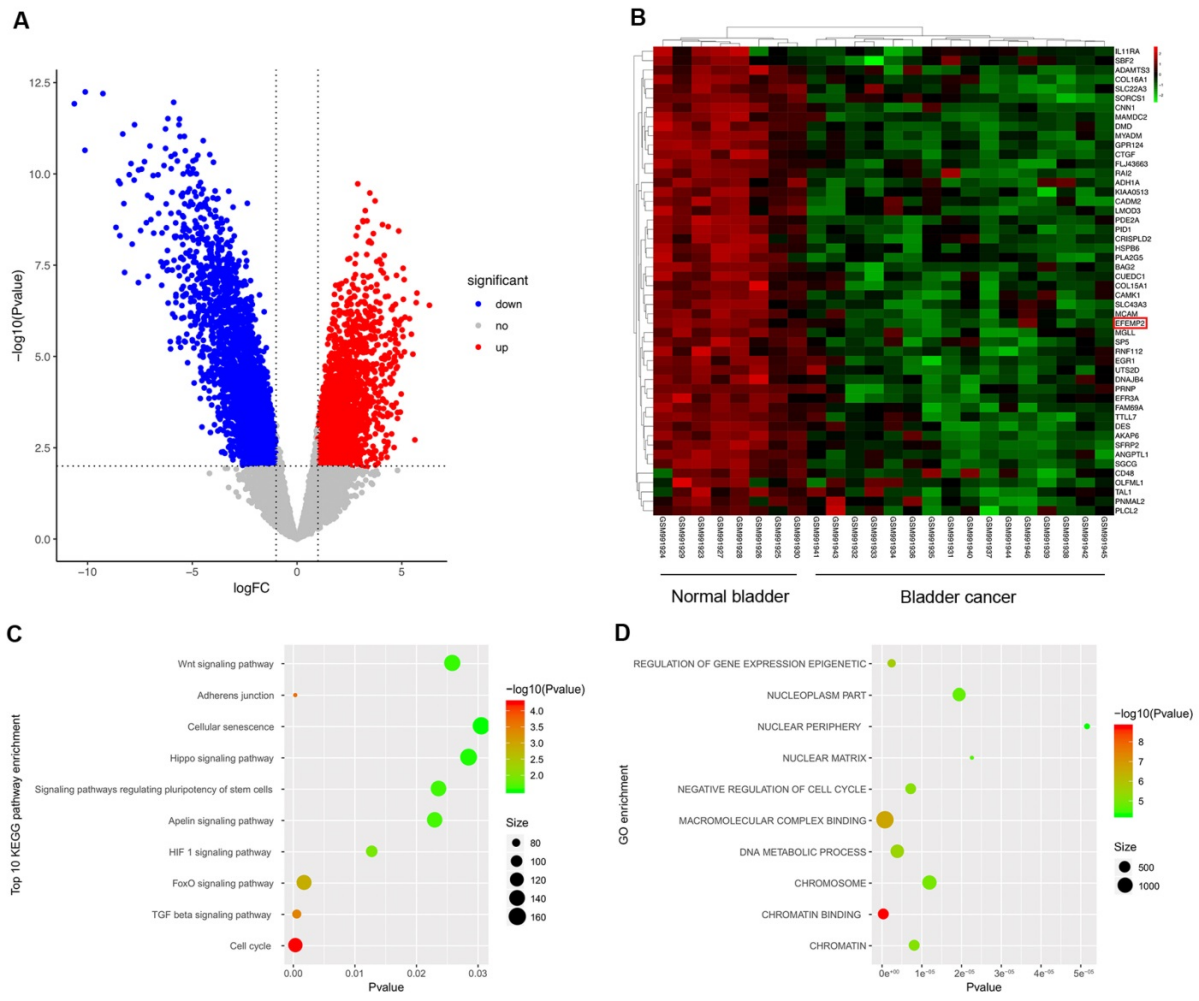
** Differentially expressed genes in GSE40355 dataset, and pathway enrichment of EFEMP2. (A)** Volcano plot visualizing the all differentially expressed genes in GSE40355,** (B)** Heatmap was used to show the unsupervised clustering of the top 50 down-regulated RNAs.** (C and D)** KEGG pathway enrichment and GO enrichment of EFEMP2 in miRNA Cancer MAP database.

All of the 24 specimens consist of 16 BC specimens and 8 non-tumor bladder specimens in the data set. Compared to normal tissues, a total of 5442 differentially expressed RNAs were screened out in BCa samples, including 2524 up-regulated RNAs and 2918 down-regulated ones. Volcano plot (Fig. 1A) was performed to represent the significantly different RNAs between bladder cancer specimens and adjacent non-cancer specimens, Heatmap was used to show the unsupervised clustering of top 50 down-regulated RNAs, and EFEMP2 is one of the members (Fig. 1B). To further explore the possible functions of EFEMP2, we analyzed function enrichment with the miRNA Cancer MAP database (*http://cis.hku.hk/miRNACancerMAP/index.php*). The results presented that EFEMP2 was strongly enriched in several pathways, such as Wnt signaling pathway, ahherens junction, and FoxO signaling pathway (Fig. 1C-D).

### Downregulation of EFEMP2 in BCa tissues and cells

**Figure 2:**
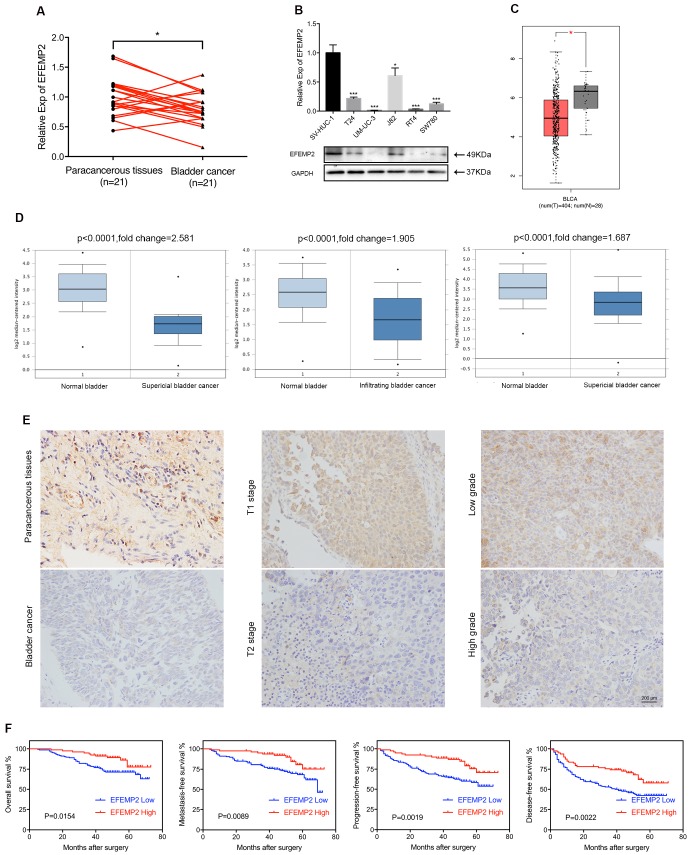
** The expression of EFEMP2 was significantly upregulated in normal bladder tissues and cell line, the expression of EFEMP2 was negatively associated with tumor stage and tumor grade of BCa, and low EFEMP2 expression was a high-risk predictor of BCa survival. (A)** qRT-PCR analysis demonstrated that EFEMP2 expression was obvious higher in normal bladder tissues compared with those in BCa tissues. **(B)** qRT-PCR and Western blot analyses indicated that the expression level of EFEMP2 in normal bladder cell line (SV-HUC-1) and BCa cell lines (T24, UM-UC-3, J82, RT4 and SW780). **(C)** EFEMP2 expression was greatly high in normal bladder tissues in GEPIA database. **(D)** The expression of EFEMP2 in the Oncomine database. Three microarray data described a strong upregulation of EFEMP2 in normal bladder tissues compared with those in both superficial and infiltrating BCa tissues. The p value and fold changes are represented. **(E)** IHC analysis also showed EFEMP2 expression was obvious higher in normal bladder tissues compared with those in BCa tissues, and EFEMP2 expression was relatively high in low tumor stage and low tumor grade. **(F)** The patients with high EFEMP2 expression had a superior OS, MFS, PFS and DFS. *p<0.05, **p<0.01, ***p<0.001.

qRT-PCR was applied to evaluate the expression level of EFEMP2 mRNA in BCa, presenting that in normal bladder tissues EFEMP2 mRNA was significantly higher than BCa tissues (Fig. 2A). The same result was found in the BCa cell lines that both transcription and translation level of EFEMP2 was increased in bladder urothelial cell line SV-HUV-1 compared with T24, UM-UC-3, J82, RT4 and SW780 cell lines (Fig. 2B). And the IHC staining results (Fig. 2E) turned out the protein level of EFEMP2 was upgraded in normal bladder tissues. In addition, the results of EFEMP2 transcriptional level in GEPIA database (*www.gepia.cancer-pku.cn*, Fig. 2C) and Oncomine database (*www.oncomine.org*, Fig. 2D) had further confirmed our findings.

### EFEMP2 could be a prognostic biomarker for BCa

In terms of the results of IHC staining, the high expression intensity of EFEMP2 in 231 BCa patients was 35.06% (81 of 231), in contrast, the EFEMP2 high staining was 66.67% (26 of 39) in paracancerous specimens (p=0.031, Fig. 2E). And as shown in Table 1, EFEMP2 expression was negatively associated with tumor stage (p=0.016, Fig. 2E) and tumor grade (p=0.026) in BCa. However, little relationship was found between EFEMP2 expression and other clinical features such as patient's gender (p=0.578), age (p=0.594), tumor size (p=0.259), multiplicity of tumor (p=0.838) and smoking history (p=0.876).

To further elucidate the role of EFEMP2 expression in BCa, the overall, metastasis-free, progress-free and disease-free survival of 222 BCa patients (2 patients in high EFEMP2 group and 7 patients in low EFEMP2 group were lost our contact) were evaluated by the Kaplane-Meier method. Follow-up for all patients included regular cystoscopy or CT scan for evaluating postoperative recurrence, the deadline for follow-up is the end of December 2016. During the period of follow-up, 14.1% (11 of 78) of patients with high EFEMP2 expression (median follow-up was 50 months, ranged 13 to 73 months) eventually died, whereas 29.2% (42 of 144) of patients died in those patients with low EFEMP2 expression (median follow-up was 49 months, ranged 5 to 72 months); and 33.3% (26 of 78, including 6 dead patients) of patients experienced tumor recurrence in high EFEMP2 group, 54.8% of patients (79 of 144, including 32 dead patients) in low EFEMP2 group; 19.2% of patients (15 of 78, including 5 dead patients) had progressed in high EFEMP2 arm versus 39.6% of patients (57 of 144, including 32 dead patients) in low EFEMP2 set; 14.1% of patients (11 of 78, including 5 dead patients) and 31.3% of patients (45 of 144, including 30 dead patients) developed metastatic disease in high and low expression of EFEMP2 respectively. The analyses depicted that the patients with high EFEMP2 expression presented a superior overall (p=0.015), metastasis-free (p=0.009), progress-free (p=0.002) and disease-free (p=0.002) survival rates compared with those patients with low EFEMP2 expression (Fig. 2F).

As showed in the Table 2, the univariate analysis described that tumor stage, tumor grade and low EFEMP2 expression significantly affected OS in BCa patients, after incorporating these factors into multivariate analysis, it described that tumor stage (HR: 2.199; 95% CI, 1.158-4.175; p=0.016) and low EFEMP2 expression (HR:1.962; 95% CI, 1.004-3.833; p=0.049) impacted the OS. The univariate analysis also represented that tumor stage, tumor grade and low EFEMP2 expression interfered MFS in BCa patients, and the multivariate analysis revealed that the tumor stage (HR: 1.950; 95% CI, 1.030-3.691; p=0.040) and low EFEMP2 expression (HR:2.065; 95% CI, 1.062-4.016; p=0.033) were still independently associated with MFS. And as presented in the Table 3, the univariate analysis also showed that tumor stage, tumor grade and low EFEMP2 expression were the obvious prognostic factors for PFS, and the multivariate analysis demonstrated that tumor stage (HR: 2.684; 95% CI, 1.615-4.460; p<0.001) and low EFEMP2 expression (HR:2.206; 95% CI, 1.243-3.915; p=0.007) were the independent prognostic factors of PFS. Moreover, tumor grade (HR:2.285; 95% CI, 1.552-3.363; p<0.001) and low EFEMP2 expression (HR:1.825; 95% CI, 1.169-2.849; p=0.008) were the prognostic factors for DFS.

### Effects of EFEMP2 on BCa cells proliferation, viability and migration in vitro

**Figure 3:**
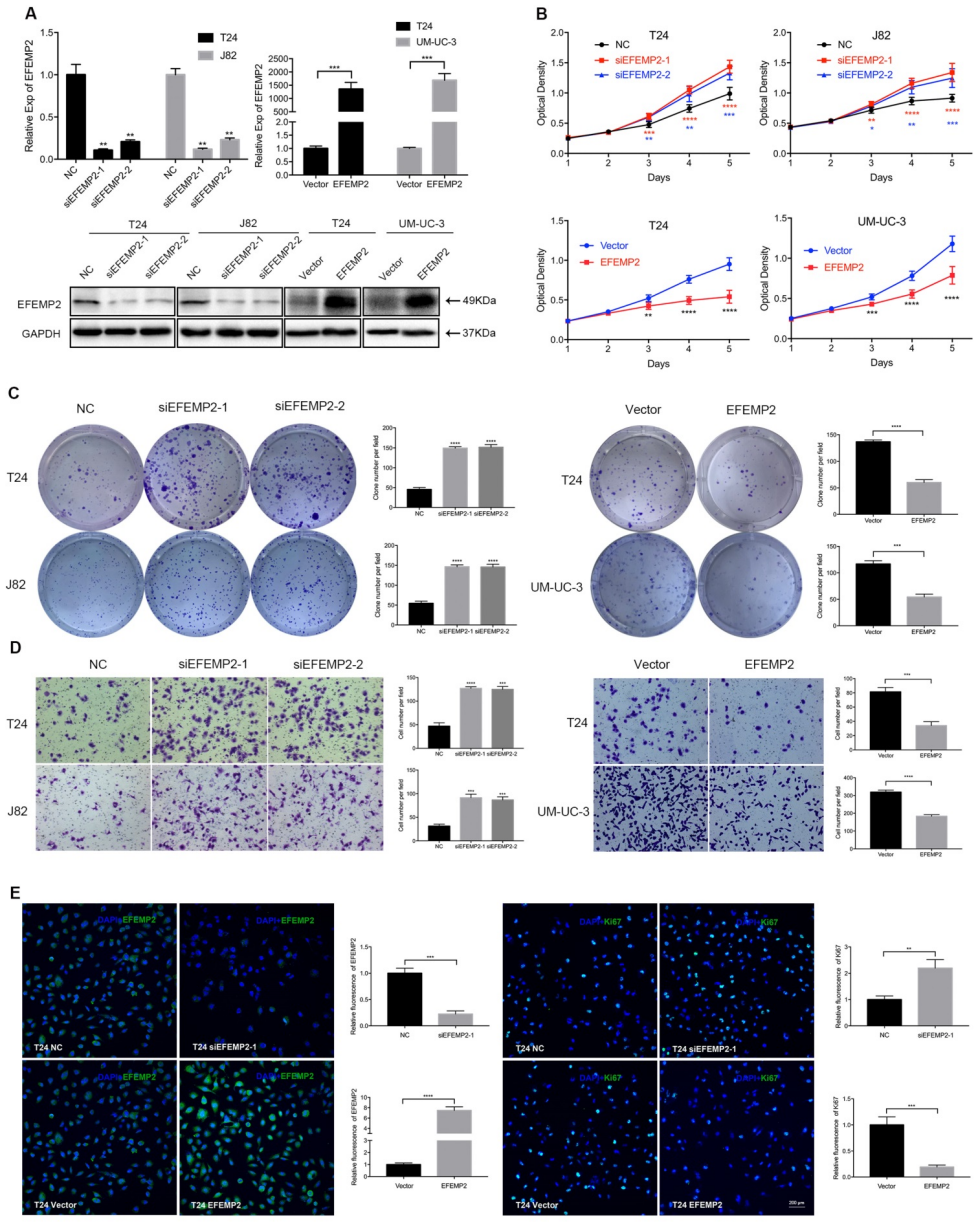
** Increased expression of EFEMP2 attenuated BCa cells proliferation and migration in vitro. (A)** qRT-PCR and Western blot analyses affirmed the interfering and upregulated efficiency of EFEMP2 in relevant BCa cells (T24, J82 and UM-UC-3). **(B)** The MTT assay assessed the ability of proliferation and viability in EFEMP2-silencing and EFEMP2-overexpression BCa cells. **(C)** Clonogenic survival assay estimated the capacity of colony formation in EFEMP2-silencing and EFEMP2-overexpression BCa cells and the clone number was statistically analyzed. **(D)** The transwell migration assay calculated the migration ability in EFEMP2-silencing and EFEMP2-overexpression BCa cells and the cell number was statistically analyzed. **(E)** Representative EFEMP2 (green) and Ki67 (green) staining in EFEMP2-silencing and EFEMP2-overexpression BCa cells. Nuclei are counterstained by DAPI (blue). *p<0.05, **p<0.01, ***p<0.001, ****p<0.0001.

The above results suggested that EFEMP2 could be a prognostic biomarker for BCa patients, patients with high expression of EFEMP2 had a superior survival. To further explore the mechanism how EFEMP2 influences the biological behavior of BCa, we conducted related experiments *in vitro* and *in vivo*. The cell model of EFEMP2 deficiency (T24 and J82) and EFEMP2 overexpression (T24 and UM-UC-3) were established by transfecting EFEMP2-target-specific-siRNA and 2xFIag-pcDNA3-EFEMP2 vector respectively. To confirm the knockdown and overexpression efficiency, the qRT-PCR and Western blot analyses (Fig. 3A) were carried out after 48 hours transfection. The MTT assay demonstrated that deficiency of EFEMP2 dramatically promoted the proliferation and viability of BCa cells, by contrary, the overexpression of EFEMP2 significantly induced the inhibition of cell growth (Fig. 3B). In line with its suppressed proliferation role, clonogenic survival assay suggested that the colony formation capacity in the EFEMP2-silencing cells were drastically improved, while obviously restrained in EFEMP2 overexpression cells (Fig.3C). And as showed in the results of transwell migration (Fig. 3D), it uncovered that decreased EFEMP2 expression restrained the migratory capability of both T24 and J82 cell lines, while upregulated EFEMP2 expression facilitated the migratory ability of both T24 and UM-UC-3 cell lines. In addition, immunofluorescence staining was applied to evaluate Ki67, a cell proliferation indicator, a similar conclusion was draw out (Fig. 3E). However, as showed in Supplementary Figure S1, knockdown or upregulation of EFEMP2 did not induce the change of cell cycle in BCa cells.

### Upregulation of EFEMP2 inhibits BCa growth and pulmonary metastasis *in vivo*

**Figure 4:**
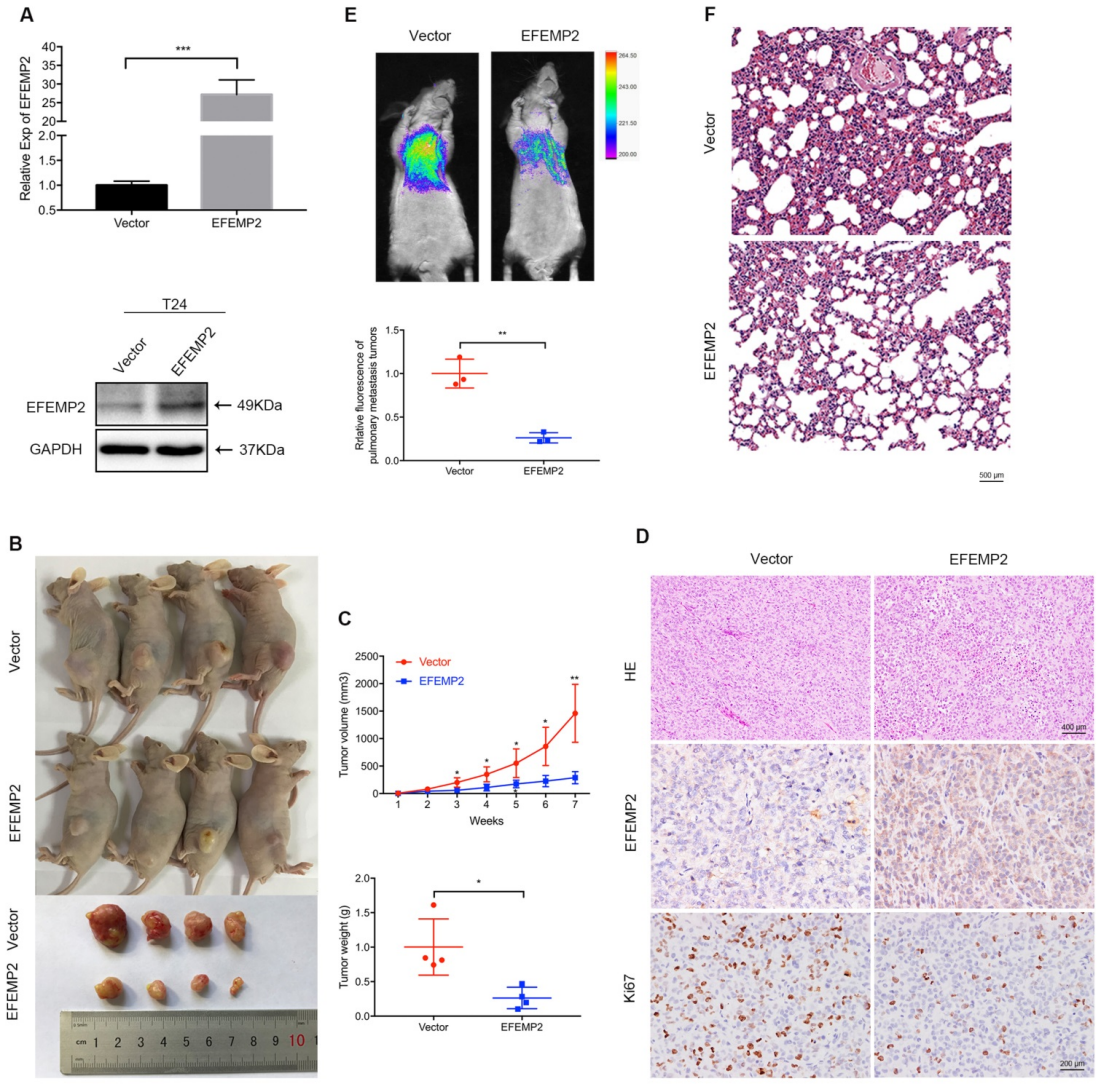
** Increased expression of EFEMP2 attenuated BCa cells proliferation and migration in vivo. (A)** qRT-PCR and Western blot analyses verified the overexpressed efficiency of EFEMP2 in stable cells (T24). **(B)** Mouse xenograft model was observed continuously for 7 weeks until the mice were killed and the tumors were dissected and weighed. **(C)** Statistical analysis of tumor volume and tumor weight. **(D)** HE staining was used to detect the nucleus atypia of tumors and IHC staining was applied to assess the expression of EFEMP2 and Ki67. **(E)** Pulmonary metastasis model, after injection for 5 weeks, the fluorescence of BCa cells pulmonary metastasis was observed to evaluate the migration capacity and statistical analysis of the fluorescence intensity was carried. Then the lungs of mice were isolated. **(F)** HE staining was utilized to indicate the pulmonary metastasis cells of mice lungs. *p<0.05, **p<0.01, ***p<0.001.

To evaluate the role of EFEMP2 overexpression in BCa tumorigenesis *in vivo*, lentiviral-EFEMP2-oeRNA and lentiviral-vector-oeRNA were transfected into T24 cells to establish a stable cell line. The stable cells were assessed by qRT-PCR and Western blot to validate the efficiency of EFEMP2 overexpression (Fig. 4A). Then T24 LV-V cells or T24 LV-EFEMP2 oe cells were subcutaneously injected into BALB/c-un mice to construct the xenograft model. As presented in Figure 4B, the EFEMP2 overexpression group had a slower growth of neoplasms compared with those empty vector group, and the average weight of tumors was light than the empty vector group (Fig. 4B). The dissected tumors then analyzed using HE and IHC staining, the HE staining results suggested that the EFEMP2 overexpression group has a lower degree of nucleus atypia, the IHC staining showed that the expression of EFEMP2 in LV-V group was weak, while the expression of Ki67 in LV-EFEMP2 oe was strong in LV-V group (Fig. 4C).

For the pulmonary metastasis model, T24 LV-V or LV-EFEMP2 oe cells were injected into the tail vein of mice, and the fluorescence of BCa cells pulmonary metastasis was accessed to evaluate the migration and metastasis ability after 1 month. The significant stronger fluorescence in the EFEMP2 overexpression group indicated that overexpression of EFEMP2 reduced cell migration ability (Fig. 4D). The HE staining results of mice lungs further confirmed the less BCa cells in EFEMP2 overexpression group (Fig. 4E).

### Effects of EFEMP2 on EMT related genes

**Figure 5:**
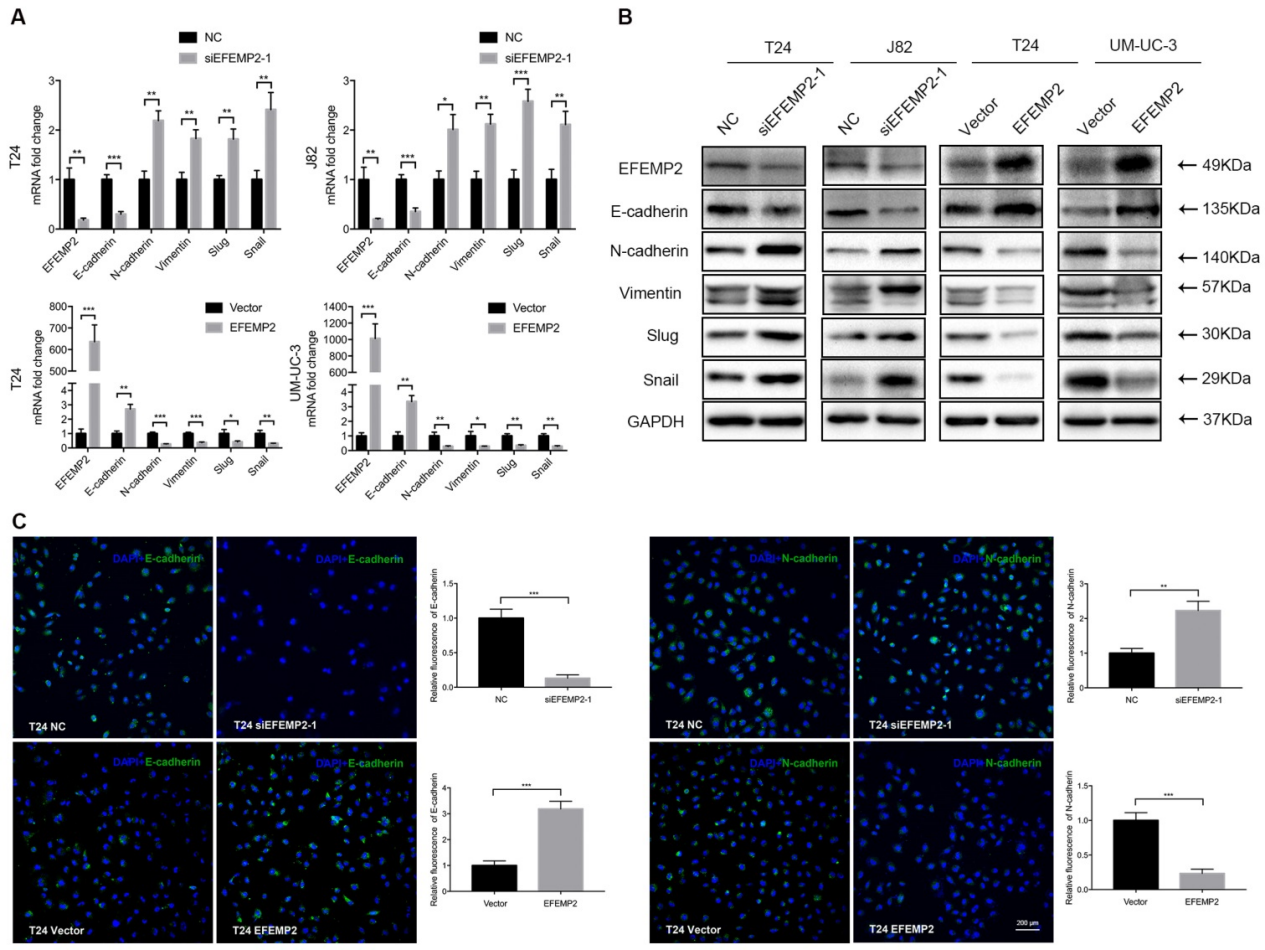
** Effects of EFEMP2 on EMT markers. (A)** The mRNA expression levels of EFEMP2 and EMT markers (E-cadherin, N-cadherin, vimentin, slug and snail) in EFEMP2-silencing and EFEMP2-overexpression BCa cells were estimated by qRT-PCR. **(B)** The protein levels of EFEMP2 and EMT markers (E-cadherin, N-cadherin, vimentin, slug and snail) in EFEMP2-silencing and EFEMP2-overexpression BCa cells were estimated by Western blot. **(C)** Immunofluorescence staining was used to further detected the expression of E-cadherin and N-cadherin in EFEMP2-silencing and EFEMP2-overexpression T24 cells. *p<0.05, **p<0.01, ***p<0.001.

It is acknowledged that EMT is highly associated with tumor invasion and metastasis, which cells acquire exercise capacity from the EMT process with phenotype changes (epithelial-like type to a mesenchymal- like type). Meanwhile, EFEMP2 also play a certain role in tumor invasion and metastasis. Thus, we suspected that EFEMP2 could exert an effect on the EMT related proteins. To clarify whether knockdown and upregulation EFEMP2 induced EMT process, several key EMT related factors including E-cadherin, N-cadherin, vimentin, snail and slug were detected both on mRNA and protein expression level. It turned out that knockdown of EFEMP2 could significantly reduce the epithelial marker E-cadherin, and increase mesenchymal markers N-cadherin, vimentin, snail and slug; reversely, EFEMP2 overexpression obviously increased E-cadherin expression, and decreased N-cadherin, vimentin, snail and slug expression (Fig. 5A and 5B). Additionally, immunofluorescence staining of E-cadherin and N-cadherin revealed a parallel result (Fig. 5C). In all, upregulated of EFEMP2 expression could suppress the migration and metastasis of BCa cells by deterring the expression of EMT related genes.

### Effects of EFEMP2 on Wnt/β-catenin signaling pathway

Wnt/β-catenin signaling pathway is prevalent in tumor invasion and metastasis, anomalous activation of the Wnt/β-catenin signaling pathway could induce the EMT process and decrease the expression of E-cadherin. We supposed that EFEMP2 may facilitate EMT action via Wnt/β-catenin signaling pathway. To investigate the presumption, both transcription and translation levels of β-catenin and Wnt pathway target genes c-Myc and cyclin D1 were ascertained.

**Figure 6:**
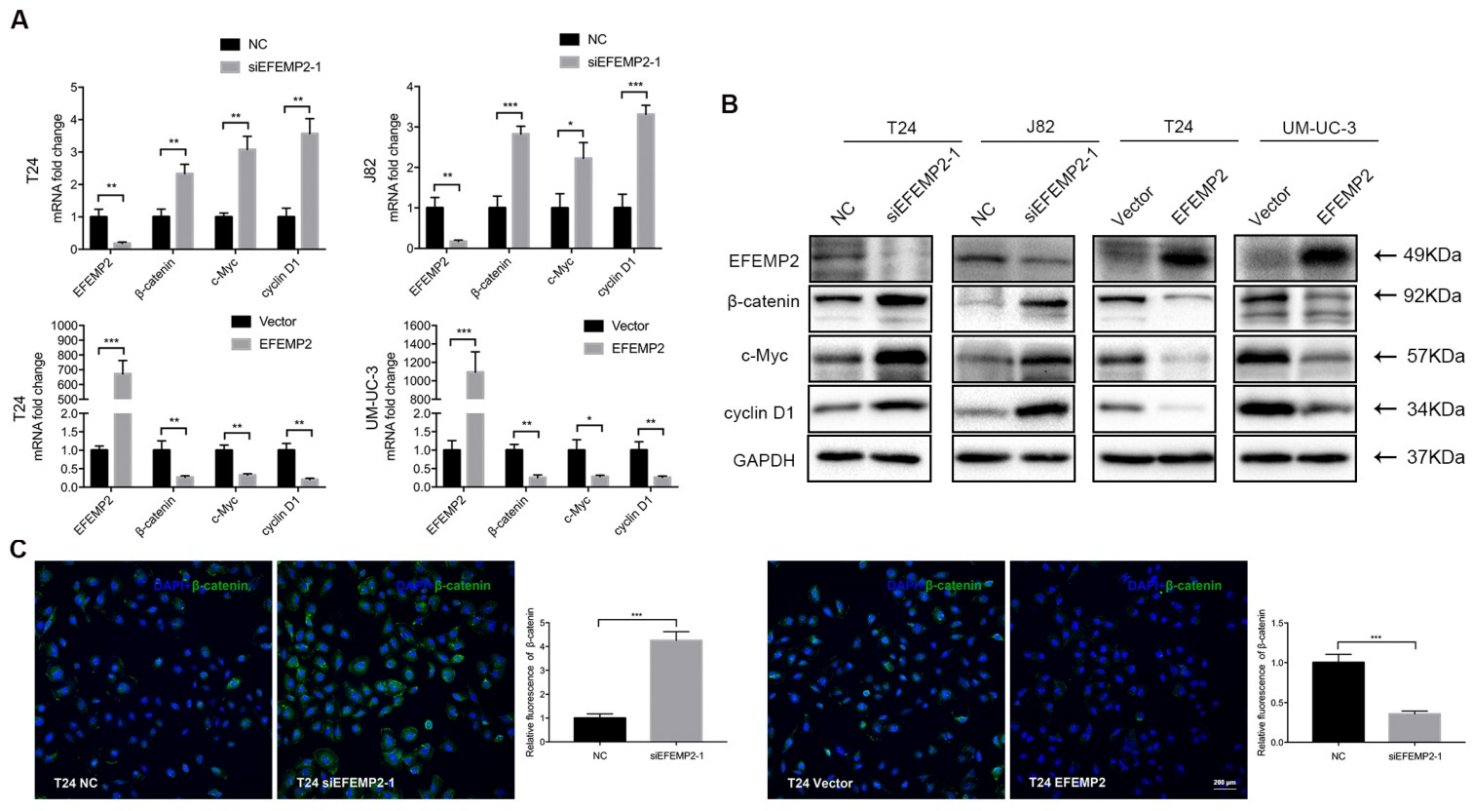
** Effects of EFEMP2 on Wnt/β-catenin signaling pathway. (A)** The mRNA expression levels of EFEMP2 and Wnt/β-catenin signaling pathway related targets (β-catenin, c-Myc and cyclin D1) in EFEMP2-silencing and EFEMP2-overexpression BCa cells were estimated by qRT-PCR. **(B)** The protein levels of EFEMP2 and and Wnt/β-catenin signaling pathway related targets (β-catenin, c-Myc and cyclin D1) in EFEMP2-silencing and EFEMP2-overexpression BCa cells were estimated by Western blot. **(C)** Immunofluorescence staining was used to further detected the expression of β-catenin in EFEMP2-silencing and EFEMP2-overexpression T24 cells. *p<0.05, **p<0.01, ***p<0.001.

We observed that the expression of β-catenin strongly increased, which was consistent with the upregulated of c-Myc and cyclin D1 in EFEMP2-silenceing cells. In addition, as we expected, the expression level of β-catenin, c-Myc and cyclin D1 were decreased in EFEMP2 overexpression cells compared with empty vector cells (Fig. 6A-B). The immunofluorescence staining of β-catenin obtained a similar ending (Fig. 6C).

### EFEMP2 inhibits EMT in BCa through the Wnt/β-catenin pathway

**Figure 7:**
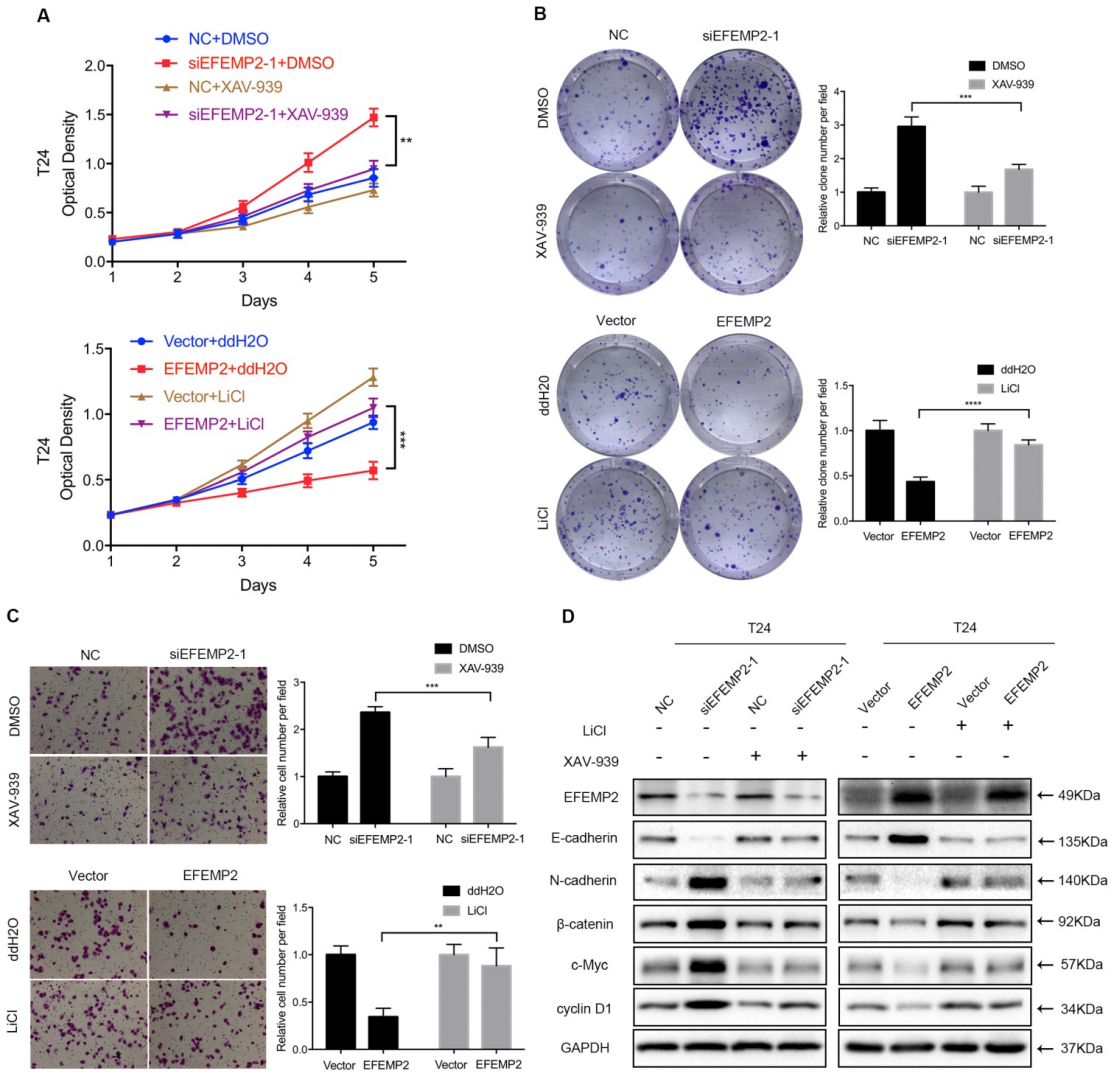
** EFEMP2 inhibited EMT through Wnt/β-catenin signaling pathway. (A-C)** Rescue experiments of siEFEMP2 and oeEFEMP2 by using XAV939 or LiCl treated (either 10 μmol/l XAV939 or 20 mmol/l LiCl for 24h): MTT, clonogenic survival and transwell migration assay. **(D)** The protein levels of EFEMP2, EMT markers (E-cadherin and N-cadherin) and Wnt/β-catenin signaling pathway related targets (β-catenin, c-Myc and cyclin D1) in EFEMP2-silencing and EFEMP2-overexpression T24 cells (either 10 μmol/l XAV939 or 20 mmol/l LiCl for 24h) were assessed by Western blotting. **p<0.01, ***p<0.001, ****p<0.0001.

In the previous experiments, it was turned out that the knockdown and overexpression of EFEMP2 could induce the changes of related markers EMT and key factors of Wnt/β-catenin pathway. To further explore whether EFEMP2 inhibits EMT process via the Wnt/β-catenin pathway, both the Wnt/β-catenin signaling-specific inhibitor XAV-939 and Wnt/β-catenin activator LiCl were utilized to rule the Wnt/β-catenin signaling in T24 cells. As showed in MTT assay (Fig. 7A), the XAV-939 could significantly rescue the promotion of proliferation and viability in EFEMP2-silencing BCa cells, while LiCl could obviously retrieval the inhibition of proliferation and viability in EFEMP2 overexpression cells. The same results were exhibited in the clonogenic survival and transwell migration assay (Fig. 7B-C). Furthermore, we also observed that changes of EMT markers expression (E-cadherin, N-cadherin) and Wnt/β-catenin factors (β-catenin, c-Myc, cyclin D1) reasoned by EFEMP2 interfering or upregulation can be recovered by XAV939 or LiCl, respectively (Fig. 7D). Collectively, these results highly demonstrated that EFEMP2 possibly prevented EMT process through Wnt/β-catenin pathway.

EFEMP2 increase degradation velocity of key proteins in Wnt/β-catenin signaling pathway in BCa cells and EFEMP2 expression is negatively correlated with the expression of Wnt/β-catenin signaling pathway related genes in human BCa specimens (n = 25).

**Figure 8:**
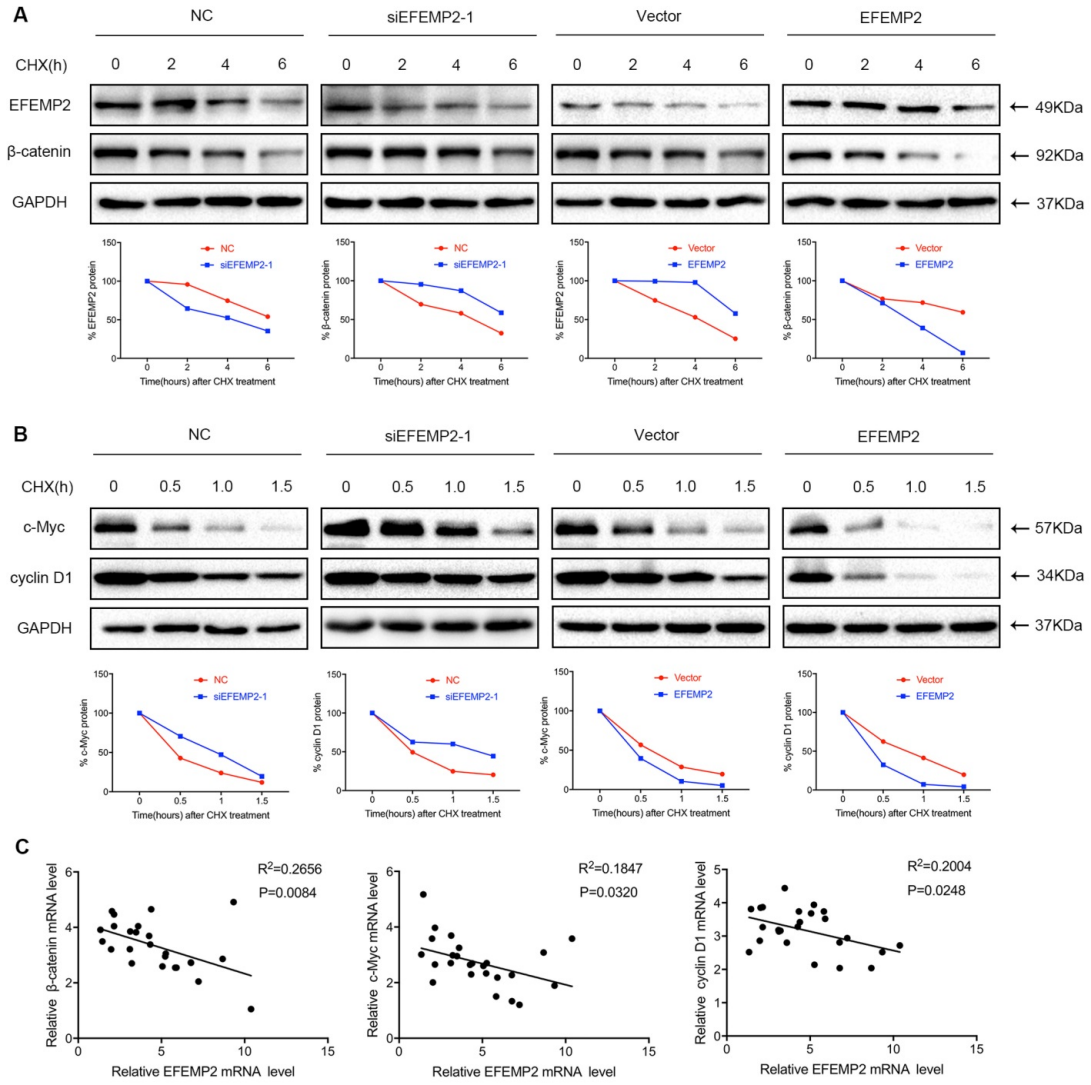
** EFEMP2 increase velocity of β-catenin, c-Myc and cyclin D1 degradation in BCa cells and EFEMP2 expression is negatively correlated with the expression of Wnt/β-catenin signaling pathway related genes in human BCa specimens (n = 25). (A-B)** Velocity of β-catenin, c-Myc and cyclin D1 degradation was measured in a time-course experiment by western blot after protein synthesis inhibition with cycloheximide (100 μg/ml) in siRNA-control-, siEFEMP2-, vector- or EFEMP2-transfected T24 cells. **(C)** The scatter plots represent the levels of EFEMP2 and Wnt/β-catenin signaling pathway markers and determined by qRT-PCR. The correlation coefficient (R^2^) and p values as calculated by linear regression (95% confidence interval) are shown inside each panel.

Moreover, a cycloheximide study demonstrated that upregulation of EFEMP2 increased velocity of β-catenin, c-Myc and cyclin D1 degradation in BCa cells, whereas the knockdown of EFEMP2 decreased degradation velocity of β-catenin, c-Myc and cyclin D1 (Fig. 8A-B). As EFEMP2 could restrain Wnt/β-catenin signaling pathway in current study, we further to examine whether the negative correlation between the expression of EFEMP2 and Wnt/β-catenin signaling pathway-related genes can be established in human BCa specimens, the expression of EFEMP2 and Wnt/β-catenin signaling pathway genes in human BCa specimens (n = 25) were analyzed. As shown in Fig 8C, the expression of EFEMP2 was negative correlated with β-catenin, c-Myc and cyclin D1.

## Discussion

BCa is a major epidemiologic problem currently with an increasing incidence by year 22. The high rate of recurrence is the primary feature of bladder cancer that makes follow-up a crucial component in effective management 3, which made a huge challenge to the global economic 4, 5. Therefore, effective measures to accurately predict the prognosis of BCa or even prevent the tumor relapse, progression and metastasis are urgently needed for clinical management.

In the current research, we found that the expression of EFEMP2 in normal bladder tissues and cells were significantly higher than those in cancer tissues and cells. Furthermore, the high expression of EFEMP2 is negatively correlated with the high stage and high grade of BCa. The survival of patients with high expression of EFEMP2 was also superior than those of patients with low expression, and the multivariate analysis demonstrated that low EFEMP2 expression were the independent poor prognostic factors of OS, MFS, PFS and DFS. It reveals that EFEMP2 plays an important role in the formation and development of BCa.

EFEMP2 belongs to the fibulin family, which composed of a group of extracellular matrix proteins with similar structures 6, 7. Studies have shown that fibulins play an important role in regulating cell morphology, proliferation, adhesion and metastasis 8. Recently, it has been reported that EFEMP2 may also participate in carcinogenesis by regulating EMT and this could be a mechanism EFEMP2-mediated cancer invasion and metastasis. Zhang et al. 23 revealed that the expression of EFEMP2 was obviously upregulated in osteosarcoma samples and strong-invasive-ability cell lines, but not in normal tissues and weak-invasive-ability cell lines, and EFEMP2-overexpressing osteosarcoma cells obtained an increased proliferation, invasion and metastasis capability by enhanced the EMT process. However, the mechanism of EFEMP2 in BCa cell invasion, and metastasis still remains uncovered.

Distant metastasis of tumor is the major factor of death in BCa and often means therapeutic failure in patients 24, 25. Therefore, it is particularly necessary to explore the mechanism of tumor invasion and metastasis. Migration as the first cascade of metastasis is the main manifestation of tumor metastasis. EMT process enables the epithelial cells to acquire ability of migration 17, 26, 27, and EMT is an important approach for infiltration and metastasis of approximately 90% of epithelial malignant tumors in human 28. E-cadherin is the key epithelial marker of EMT which locates at the junction between epithelial cells and participates in the formation of intercellular adhesion structures. When E-cadherin is reduced, the tight links between cells become loose. The cells' adhesion ability is decreased, and cells can be easy to fall off from the primary tumor site, which increases the cells' ability to move and invade 29, 30. In this study, we found that the ability of migration and metastasis was significantly increased in EFEMP2-interfering cells, inversely decreased EFEMP2 expression restrained the capability of migration and metastasis both in vitro and in vivo. And knockdown of EFEMP2 could downregulate the expression of epithelial marker (E-cadherin) and upregulate the expression of interstitial factors (N-cadherin, vimentin, snail and slug, and EFEMP2 overexpression obviously increased epithelial factor, and reduced interstitial markers. Therefore, we supposed that EFEMP2 was involved in EMT.

Wnt/β-catenin signaling pathway has a major impact on EMT during cancer progression. Abnormal activation of the Wnt/β-catenin signaling pathway is ubiquitous in tumor tissues, and β-catenin is the central protein in this signaling pathway 19-21. Under normal circumstances, lack of Wnt signal, β-catenin and E-cadherin are attaching on the cell membrane, and very few are presenting in the cytoplasm. The loss of E-cadherin may abnormally activate the pathway, the degradation of β-catenin is inhibited, and β-catenin enters into the nucleus to promote the expression of downstream target genes such as c-Myc and cyclin D1, and finally promote the malignant progression of cells 29, 30. According to the published reports 31, 32, researches had suggested that Wnt/β-catenin signaling pathway played an important in the etiology of BCa, but more study is still needed to explore the association among EFEMP2, EMT and Wnt/β-catenin signaling pathway in BCa. The current research revealed that EFEMP2 downregulation could facilitate EMT and boost Wnt/ β-catenin signaling pathway which verified by the upregulated expression of both β-catenin and β-catenin target genes (c-Myc, cyclin D1) in EFEMP2-silencing BCa cells. Inversely, EFEMP2 overexpression could inhibit EMT and prevent Wnt/ β-catenin signaling pathway. Moreover, the cycloheximide study revealed that EFEMP2 could increase degradation velocity of β-catenin, c-Myc and cyclin D1 in BCa cells. Since EFEMP2 could restrain Wnt/β-catenin signaling pathway in current study, we further to examine whether the negative correlation between the expression of EFEMP2 and Wnt/β-catenin signaling pathway-related genes can be established in human BCa specimens and we validated the negative correlation between EFEMP2 and Wnt/β-catenin signaling pathway markers. Further studied had suggested that changes of EMT factors induced by EFEMP2 interfering or upregulation could be recovered by XAV939 or LiCl, respectively. These findings suggested that EFEMP2 potentially suppresses EMT through Wnt/β-catenin signaling pathway in BCa cells.

## Conclusions

In summary, EFEMP2 is an auspicious predictive biomarker for BCa, which may help physicians in identifying high-risk patients who might benefit from early, aggressive and tailored therapy after surgical treatment. Additionally, the current research demonstrated that EFEMP2 could act as an inhibitor of EMT by weakening the Wnt/β-catenin signaling pathway and thus plays a significant role in BCa invasion and metastasis.

## Supplementary Material

Supplementary figures and tables.

## Figures and Tables

**Table 1:** Correlation between EFEMP2 expression and clinical features of patients

Variable	Total (n=231)	EFEMP2 high (n=80)	EFEMP2 low (n=151)	P
Gender				0.578
Male (%)	177 (76.6)	63 (78.2)	114 (75.7)	
Female (%)	54 (23.4)	17 (21.8)	37 (24.3)	
Age (years)	65.4 ± 10.87*	64.9 ± 10.22	65.7 ± 11.19	0.594
Tumor size (cm)	2.5 ± 1.09	2.4 ± 0.95	2.5 ± 1.15	0.259
Tumor stage				0.016
Ta, T1 (%)	192 (82.9)	73(91.0)	119 (78.5)	
T2, T3, T4 (%)	39 (17.1)	7 (9.0)	32 (21.5)	
Tumor grade				
PUNLMP, low grade (%)	141 (63.5)	59 (73.1)	89 (58.3)	0.026
High grade (%)	81 (36.5)	21 (26.9)	62 (41.7)	
Multiplicity of tumor				0.838
No (%)	177 (79.7)	65 (80.8)	121 (79.2)	
Yes (%)	45 (20.3)	15 (19.2)	30 (20.8)	
Smoking history				0.876
Yes (%)	87 (39.2)	32 (38.5)	62 (39.6)	
No (%)	135 (60.8)	48 (61.5)	89 (60.4)	

*The value = mean ± standard deviation; PUNLMP=Papillary urothelial neoplasm of low malignant potential.

**Table 2:** Univariable and multivariable analysis for overall and metastasis-free survival of patients with bladder cancer.

Variable	Overall survival				Metastasis-free survival			
	Univariate		Multivariate		Univariate		Multivariate	
	HR (95%CI)	P	HR (95%CI)	P	HR (95%CI)	P	HR (95%CI)	P
Gender (Female vs Male)	1.150 (0.614-2.156)	0.662	-	-	1.201 (0.654-2.206)	0.555	-	-
Age	1.025 (0.998-1.053)	0.069	-	-	1.018 (0.993-1.043)	0.161	-	-
Tumor size	2.823 (1.583-5.032)	0.077	-	-	1.109 (0.878-1.399)	0.385	-	-
Tumor stage (T2, T3, T4 vs Ta, T1)	1.901 (1.496-2.416)	<0.001	2.199 (1.158-4.175)	0.016	2.852 (1.604-5.068)	<0.001	1.950 (1.030-3.691)	0.040
Tumor grade (High VS PUNLMP, Low)	1.946 (1.135-3.338)	0.016	1.405 (0.774-2.551)	0.263	2.351 (1.385-3.988)	0.002	1.768 (0.983-3.178)	0.057
Multiplicity (Yes vs No)	0.638 (0.301-1.355)	0.242	-	-	0.761 (0.383-1.513)	0.436	-	-
Smoking (yes vs no)	1.082 (0.623-1.878)	0.781	-	-	1.065 (0.621-1.826)	0.818	-	-
EFEMP2 status (Low vs High)	2.221 (1.143-4.316)	0.019	1.962 (1.004-3.833)	0.049	2.345 (1.211-4.538)	0.011	2.065 (1.062-4.016)	0.033

HR=hazard ratio; CI=confidence interval; PUNLMP=Papillary urothelial neoplasm of low malignant potential.

**Table 3:** Univariable and multivariable analysis for disease-free and progression-free survival of patients with bladder cancer.

Variable	Progression-free survival				Disease-free survival			
	Univariate		Multivariate		Univariate		Multivariate	
	HR (95%CI)	P	HR (95%CI)	P	HR (95%CI)	P	HR (95%CI)	P
Gender (female vs male)	0.847 (0.479-1.497)	0.568	-	-	1.323 (0.862-2.030)	0.201	-	-
Age	1.007 (0.986-1.028)	0.533	-	-	0.996 (0.980-1.013)	0.631	-	-
Tumor size	1.126 (0.920-1.379)	0.251	-	-	1.100 (0.927-1.307)	0.275	-	-
Tumor stage (t2, t3, t4 vs ta, t1)	1.823 (1.068-3.113)	0.028	0.978 (0.545-1.755)	0.941	0.920 (0.554-1.530)	0.749	-	-
Tumor grade (high VS PUNLMP, low)	2.818 (1.770-4.487)	<0.001	2.684 (1.615-4.460)	<0.001	2.395 (1.630-3.518)	<0.001	2.285 (1.552-3.363)	<0.001
Multiplicity (yes vs no)	0.823 (0.452-1.501)	0.526	-	-	1.457 (0.940-2.257)	0.092	-	-
Smoking (yes vs no)	1.112 (0.694-1.783)	0.658	-	-	1.003 (0.667-1.485)	0.989	-	-
EFEMP2 status (low vs high)	2.391 (1.353-4.224)	0.003	2.206 (1.243-3.915)	0.007	1.960 (1.258-3.055)	0.003	1.825 (1.169-2. 849)	0.008

HR=hazard ratio; CI=confidence interval; PUNLMP=Papillary urothelial neoplasm of low malignant potential.

## Abbreviations

BCa: bladder cancer
CI: confidence interval
CT: computed tomography
DFS: disease-free survival
EFEMP2: epidermal growth factor-containing fibulin-like extracellular matrix protein 2
EMT: epithelial-mesenchymal transition
GEO: Gene Expression Omnibus
HE: hematoxylin and eosin
HR: hazard ratio
IHC: immunohistochemical
MFS: metastasis-free survival
MTT: methyl thiazolyl tetrazolium
NCBI: National Center for Biotechnology Information
OS: overall survival
PFA: paraformaldehyde
PFS: progress-free survival
PUNLMP: papillary urothelial neoplasm of low malignant potential

## References

[1] Bray F, Ferlay J, Soerjomataram I, Siegel RL, Torre LA, Jemal A (2018). Global cancer statistics 2018: GLOBOCAN estimates of incidence and mortality worldwide for 36 cancers in 185 countries. CA Cancer J Clin.

[2] Stein JP, Skinner DG (2006). Radical cystectomy for invasive bladder cancer: long-term results of a standard procedure. World J Urol.

[3] Flaig TW, Spiess PE, Agarwal N, Bangs R, Boorjian SA, Buyyounouski MK (2018). NCCN Guidelines Insights: Bladder Cancer, Version 5.2018. J Natl Compr Canc Netw.

[4] Avritscher EBC, Cooksley CD, Grossman HB, Sabichi AL, Hamblin L, Dinney CP (2006). Clinical model of lifetime cost of treating bladder cancer and associated complications. Urology.

[5] Botteman MF, Pashos CL, Redaelli A, Laskin B, Hauser R (2003). The health economics of bladder cancer - A comprehensive review of the published literature. Pharmacoeconomics.

[6] Gallagher WM, Currid CA, Whelan LC (2005). Fibulins and cancer: friend or foe?. Trends Mol Med.

[7] Argraves WS, Greene LM, Cooley MA, Gallagher WM (2003). Fibulins: physiological and disease perspectives. EMBO Rep.

[8] Timpl R, Sasaki T, Kostka G, Chu ML (2003). Fibulins: a versatile family of extracellular matrix proteins. Nat Rev Mol Cell Biol.

[9] Kobayashi N, Kostka G, Garbe JHO, Keene DR, Bächinger HP, Hanisch FG (2007). A Comparative Analysis of the Fibulin Protein Family. J Biol Chem.

[10] Giltay R, Timpl R, Kostka G (1999). Sequence, recombinant expression and tissue localization of two novel extracellular matrix proteins, fibulin-3 and fibulin-4. Matrix Biol.

[11] Obaya AJ, Rua S, Moncada-Pazos A, Cal S (2012). The dual role of fibulins in tumorigenesis. Cancer Lett.

[12] Chen L, Sun B, Zhang S, Zhao X, He Y, Zhao S (2009). Influence of microenvironments on microcirculation patterns and tumor invasion-related protein expression in melanoma. Oncol Rep.

[13] Chen J, Zhang J, Liu X, Fang R, Zhao Y, Ma D (2014). Overexpression of fibulin-4 is associated with tumor progression and poor prognosis in patients with cervical carcinoma. Oncol Rep.

[14] Motalebzadeh J, Mahjoubi F, Nafissi N, Hashemian M, Taheri M, Hosseinpour Y (2017). FBLN-4 and BCRP genes as two prognostic markers are downregulated in breast cancer tissue. Cancer Biomark.

[15] Acloque H, Adams MS, Fishwick K, Bronner-Fraser M, Nieto MA (2009). Epithelial-mesenchymal transitions: the importance of changing cell state in development and disease. J Clin Invest.

[16] Thiery JP (2002). Epithelial-mesenchymal transitions in tumour progression. Nat Rev Cancer.

[17] Thiery JP, Acloque H, Huang RY, Nieto MA (2009). Epithelial-mesenchymal transitions in development and disease. Cell.

[18] Lee JM, Dedhar S, Kalluri R, Thompson EW (2006). The epithelial-mesenchymal transition: new insights in signaling, development, and disease. J Cell Biol.

[19] Kalluri R, Weinberg RA (2009). The basics of epithelial-mesenchymal transition. J Clin Invest.

[20] Thiery JP (2003). Epithelial-mesenchymal transitions in development and pathologies. Curr Opin Cell Biol.

[21] Howard S, Deroo T, Fujita Y, Itasaki N (2011). A Positive Role of Cadherin in Wnt/beta-Catenin Signalling during Epithelial-Mesenchymal Transition.

[22] Siegel RL, Miller KD, Jemal A (2018). Cancer statistics, 2018. CA Cancer J Clin.

[23] Zhang D, Wang SG, Chen J, Liu H, Lu J, Jiang H (2017). Fibulin-4 promotes osteosarcoma invasion and metastasis by inducing epithelial to mesenchymal transition via the PI3K/Akt/mTOR pathway. Int J Oncol.

[24] Zargar-Shoshtari K, Zargar H, Lotan Y, Shah JB, van Rhijn BW, Daneshmand S (2016). A multi-Institutional analysis of outcomes of patients with clinically node positive urothelial bladder cancer treated with induction chemotherapy and radical cystectomy. J Urol.

[25] Fajkovic H, Cha EK, Jeldres C, Robinson BD, Rink M, Xylinas E (2013). Extranodal extension is a powerful prognostic factor in bladder cancer patients with lymph node metastasis. Eur Urol.

[26] Grunert S, Jechlinger M, Beug H (2003). Diverse cellular and molecular mechanisms contribute to epithelial plasticity and metastasis. Nat Rev Mol Cell Biol.

[27] Huber MA, Kraut N, Beug H (2005). Molecular requirements for epithelial-mesenchymal transition during tumor progression. Curr Opin Cell Biol.

[28] Hugo H, Ackland ML, Blick T, Lawrence MG, Clements JA, Williams ED (2007). Epithelial-mesenchymal and mesenchymal-epithelial transitions in carcinoma progression. J Cell Physiol.

[29] Wijnhoven BPL, Dinjens WNM, Pignatelli M (2000). E-cadherin-catenin cell-cell adhesion complex and human cancer. Br J Surg.

[30] MacDonald BT, Tamai K, He X (2009). Wnt/beta-Catenin Signaling: components, mechanisms, and diseases. Dev Cell.

[31] Xie X, Pan J, Han X, Chen W (2019). Downregulation of microRNA-532-5p promotes the proliferation and invasion of bladder cancer cells through promotion of HMGB3/Wnt/beta-catenin signaling. Chem Biol Interact.

[32] Gao L, Xu F, Shi W, Zhang S, Lu YL, Zhao DK (2018). High-glucose promotes proliferation of human bladder cancer T24 cells by activating Wnt/β-catenin signaling pathway. Eur Rev Med Pharmacol Sci.

