# EFEMP2 suppresses epithelial-mesenchymal transition via Wnt/β-catenin signaling pathway in human bladder cancer: Erratum

**DOI:** 10.7150/ijbs.75156

**Published:** 2022-05-27

**Authors:** Qiang Zhou, Song Chen, Mengxin Lu, Yongwen Luo, Gang Wang, Yu Xiao, Lingao Ju, Xinghuan Wang

**Affiliations:** 1 Department of Urology, Zhongnan Hospital of Wuhan University, Wuhan, China; 2 Department of Biological Repositories, Zhongnan Hospital of Wuhan University, Wuhan, China; 3 Human Genetics Resource Preservation Center of Hubei Province, Wuhan, China; 4 Laboratory of Precision Medicine, Zhongnan Hospital of Wuhan University, Wuhan, China; 5 Medical Research Institute, Wuhan University, Wuhan, China; 6 Urological Clinical Research Center of Laparoscopy in Hubei Province, Wuhan, China

In Figure 3E, the panel of “T24 NC” was exactly the same as “T24 Vector”. We apologize for our careless work, and we immediately self-surveyed and carefully rechecked the raw data. We found that we made a mistake of “copying-and-pasting” in the process of making the diagram. The panel of “T24 NC” is correct, while the panel of “T24 Vector” is pasted incorrectly in Figure 3E. We mistakenly copied and pasted figure “T24 NC” into “T24 Vector”. The correct version of Figure 3 is listed below.

We sincerely apologize for any confusion caused by our negligence and this unintentional error. The authors confirm that the corrections made in this erratum do not affect the original conclusions.

## Figures and Tables

**Figure 3:**
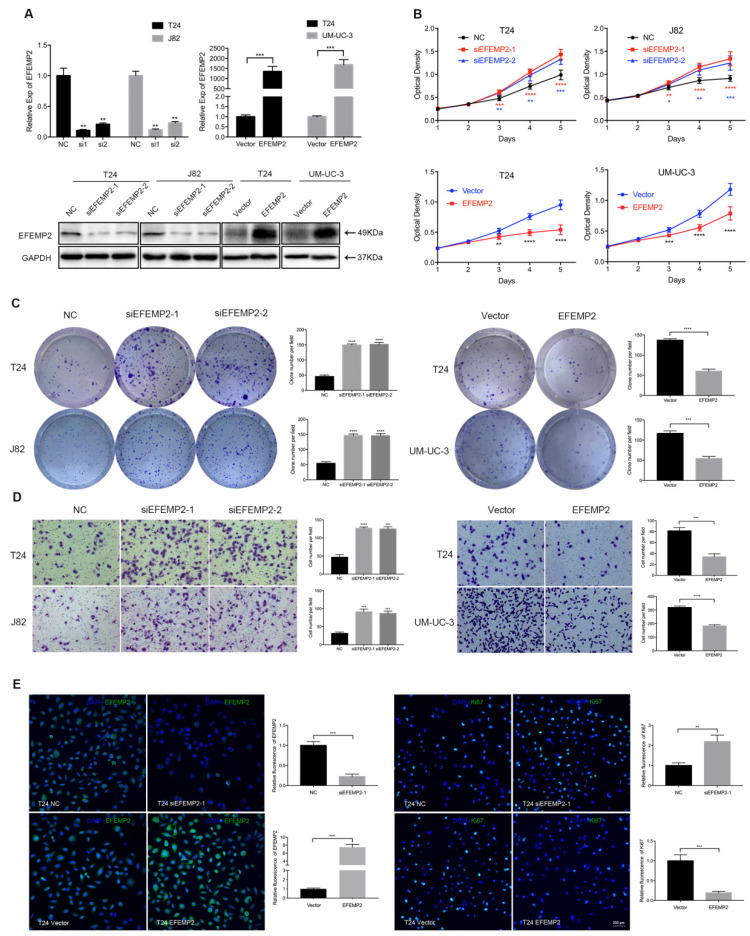
** Increased expression of EFEMP2 attenuated BCa cells proliferation and migration in vitro. (A)** qRT-PCR and Western blot analyses affirmed the interfering and upregulated efficiency of EFEMP2 in relevant BCa cells (T24, J82 and UM-UC-3). **(B)** The MTT assay assessed the ability of proliferation and viability in EFEMP2-silencing and EFEMP2-overexpression BCa cells. **(C)** Clonogenic survival assay estimated the capacity of colony formation in EFEMP2-silencing and EFEMP2-overexpression BCa cells and the clone number was statistically analyzed. **(D)** The transwell migration assay calculated the migration ability in EFEMP2-silencing and EFEMP2-overexpression BCa cells and the cell number was statistically analyzed. **(E)** Representative EFEMP2 (green) and Ki67 (green) staining in EFEMP2-silencing and EFEMP2-overexpression BCa cells. Nuclei are counterstained by DAPI (blue). *p<0.05, **p<0.01, ***p<0.001, ****p<0.0001.

