# Supplementary material for: EFEMP2 suppresses epithelial-mesenchymal transition via Wnt/β-catenin signaling pathway in human bladder cancer
Source: Int J Biol Sci. 2019 Aug 8;15(10):2139–55. doi: 10.7150/ijbs.35541 (PMC6775297; doi:10.7150/ijbs.35541)
Supplement: Supplementary file 1 — Supplementary figures and tables. [file ijbsv15p2139s1.pdf]

EFEMP2 suppresses epithelial-mesenchymal transition via Wnt/β-catenin signaling pathway in human bladder cancer

Qiang Zhou[#], Song Chen[#], Mengxin Lu, Yongwen Luo, Gang Wang, Yu Xiao, Lingao Ju[*], Xinghuan Wang[*]

Supplementary Figure

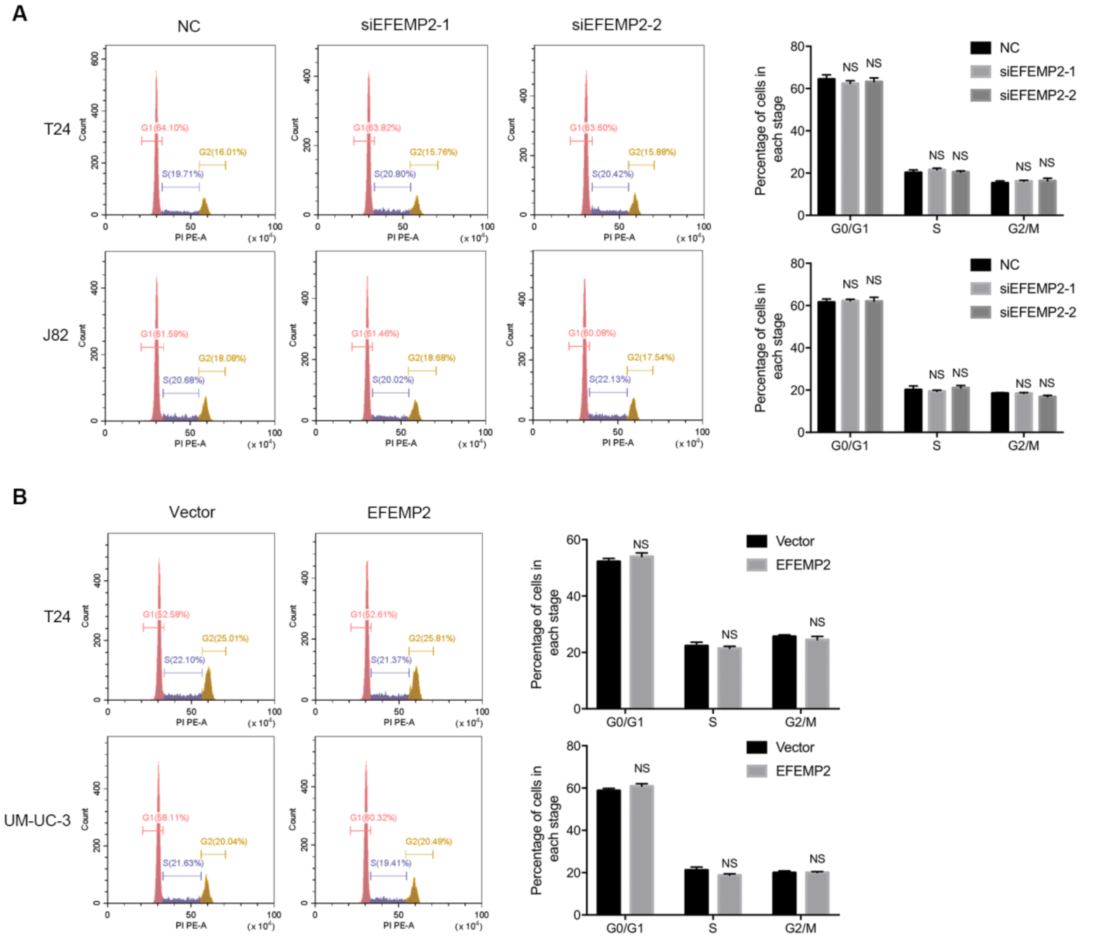

Supplementary Figure S1. Knockdown or upregulation of EFEMP2 did not induce the change of cell cycle in BCa cells. (A) Flow cytometry analysis for T24 and J82 cells treated with control-siRNA and si-EFEMP2 for 48 h. **(B)** Flow cytometry analysis for T24 and UM-UC-3 cells treated with vector and EFEMP2 for 48 h.
